# Curcumin's Therapeutic Potential in Ovarian Cancer: Current Insights and Future Perspectives

**DOI:** 10.1002/fsn3.71099

**Published:** 2025-11-28

**Authors:** Smirnova Elena, Sureshbabu Anjana, Do Thi Cat Tuong, Sungyeon Chin, Mohammad Moniruzzaman, Bui Huy Doanh, Adhimoolam Karthikeyan, Taesun Min

**Affiliations:** ^1^ Department of Animal Biotechnology, Jeju International Animal Research Center (JIA), Sustainable Agriculture Research Institute (SARI) Jeju National University Jeju Republic of Korea; ^2^ Department of Animal Biochemistry, Faculty of Animal Science Vietnam National University of Agriculture Hanoi Vietnam; ^3^ Subtropical Horticulture Research Institute Jeju National University Jeju Republic of Korea

**Keywords:** anticancer effects, gynecological cancer, natural compounds, turmeric

## Abstract

Ovarian cancer remains the leading cause of gynecological cancer‐related mortality in women, highlighting the urgent need for novel therapeutic approaches despite advances in current treatments such as chemotherapy. Curcumin, a natural compound, exhibits potent anticancer properties against ovarian cancer. This review summarizes recent advances in understanding curcumin's role in ovarian cancer therapy. Preclinical studies underscore curcumin's potential as an adjunctive agent, particularly in combination with standard therapies. It enhances treatment efficacy by sensitizing cancer cells to chemotherapy and radiotherapy. Curcumin modulates apoptotic and resistance pathways, inhibits cellular proliferation, and mitigates metastasis in ovarian cancer models. However, the transition from preclinical research to clinical application remains challenging due to the limited number of human trials evaluating curcumin's efficacy, safety profile, and optimal dosage. Recent advancements in delivery systems—such as nanoformulations and lipid‐based carriers—aim to improve curcumin's solubility and systemic absorption. These innovations not only enhance curcumin's therapeutic efficacy but also facilitate synergistic combinations with standard chemotherapeutic agents such as cisplatin, thereby improving treatment outcomes and potentially reducing adverse effects. Future research should focus on elucidating curcumin's interactions with ovarian cancer organoids and advancing multi‐omics approaches to uncover its epigenetic and therapeutic mechanisms. Addressing these challenges will support the translation of curcumin into an effective therapeutic agent for treating ovarian cancer.

## Graphical abstract

This review provides an overview of ovarian cancer, examines curcumin's emerging role in its treatment, identifies existing research gaps, and outlines a roadmap for future studies aimed at fully exploring curcumin's therapeutic potential in the management of ovarian cancer.

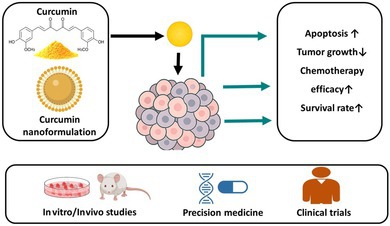

## 1 Introduction

Ovarian cancer, the second most prevalent gynecological malignancy worldwide, presents a significant global health challenge. In 2020, an estimated 313,959 new cases were diagnosed, resulting in 207,252 deaths worldwide (Sung et al. 2021). Although relatively rare compared to other cancers, ovarian cancer ranks eighth in global incidence. Notably, approximately 75% of cases are diagnosed at advanced stages, with survival rates falling below 50% (Torre et al. 2018). In South Korea, ovarian cancer accounts for 2.5% of all female cancer cases, with survival rates ranging from 95% in early‐stage diagnoses to approximately 15% in advanced stages (Korea Central Cancer Registry 2022). In the United States, an estimated 19,710 new cases and 13,270 deaths were projected for 2023, with a 5‐year relative survival rate of 50.8%. This overall figure masks significant variation by stage, with survival rates ranging from 92.4% for localized disease to 31.5% for distant metastasis (Siegel et al. 2023). Ovarian cancer is classified into three main histologic types, with epithelial ovarian cancer accounting for approximately 90% of cases, while germ and stromal cell types are less common. General symptoms of ovarian cancer often include persistent and unexplained abdominal or pelvic pain, which may vary from a dull, aching discomfort to sharp, intense pain (La Vecchia 2017; Chandra et al. 2019; Boussios et al. 2019). The precise causes of ovarian cancer remain elusive, but several identifiable risk factors have been recognized. Ovarian cancer risk rises with age, predominantly affecting postmenopausal women. Inherited genetic mutations like BRCA1 and BRCA2 significantly heighten the likelihood of developing ovarian cancer, and a family history of ovarian or breast cancer can also elevate one's risk (Lancaster et al. 2015).

The asymptomatic progression of ovarian cancer has earned it the moniker “silent killer”, reflecting the persistent challenges associated with early detection. The 5‐year survival rate drops significantly when the disease is diagnosed at an advanced stage. The clinical complexity of advanced‐stage ovarian cancer—characterized by limited treatment options and heterogeneous histological subtypes—continues to present significant obstacles to effective therapeutic intervention (Jacobs et al. 2016). The standard treatment for ovarian cancer primarily involves platinum‐based chemotherapy, often in combination with paclitaxel or the antiangiogenic agent bevacizumab. Despite its initial effectiveness, the development of resistance—particularly to bevacizumab—remains a major challenge, leading to disease recurrence or progression in 70%–80% of patients following primary treatment (Mai et al. 2022). Furthermore, anticancer drugs are associated with adverse effects, including lymphopenia, tiredness, diarrhea, fatigue, and loss of appetite. In response, increased awareness of symptoms and proactive management of risk factors, including age, genetic mutations, and hormonal therapy, are of significant importance. Personalized prognostic discussions are essential, and should consider factors such as the specific type of ovarian cancer and the patient's overall health status. Current research continues to focus on developing effective early detection methods and innovative treatment strategies to address the considerable global health challenge posed by ovarian cancer.

Amid ongoing challenges in disease management, plant‐derived bioactive compounds have emerged as promising alternatives for combating a range of conditions (Guo et al. 2018; Eze et al. 2022; Uma Reddy et al. 2022), including cancer (Redkar and Jolly 2003; Ma et al. 2021). Curcumin—a bioactive compound derived from rhizomes of turmeric ( *Curcuma longa* ), extensively investigated for its pharmaceutical properties in both in vitro and in vivo studies and clinical studies (Karthikeyan et al. 2021; Gao et al. 2022; Smirnova et al. 2023; Sureshbabu et al. 2023; Tuong et al. 2023; Lan et al. 2024). Many studies have demonstrated the biological effects of curcumin on multiple cellular functions, many of which are crucial to its anticancer activity (Patel et al. 2020; Pourbagher‐Shahri et al. 2021; Mohammadi et al. 2022). Curcumin has demonstrated efficacy in modulating signaling pathways involved in cellular proliferation, apoptosis, and angiogenesis in various malignancies (Tomeh et al. 2019; Zoi et al. 2022; Singh et al. 2023). Notably, clinical studies involving human participants have shown that curcumin is well‐tolerated, safe, and non‐carcinogenic (Gupta et al. 2013; Soleimani et al. 2018). In the context of ovarian cancer, curcumin functions as an anticancer agent by suppressing tumorigenesis, enhancing the efficacy of radiotherapy and chemotherapy, and minimizing damage to normal cells (Pourhanifeh et al. 2020). Curcumin's anticancer effects against ovarian cancer have been demonstrated in both in vitro and in vivo studies. Furthermore, nanoformulations of curcumin markedly enhance its bioavailability and therapeutic efficacy. However, clinical evidence in humans remains limited. Critical questions concerning the optimal dosage, long‐term safety, and pharmacokinetics of curcumin in ovarian cancer patients have yet to be fully resolved.

In this review, we first provide a brief overview of ovarian cancer, followed by an examination of curcumin's emerging role in its treatment. We also shed light on the existing research gaps and outline a roadmap for future studies aimed at fully harnessing curcumin's therapeutic potential in the management of ovarian cancer.

## 2 Ovarian Cancer: The Story So Far and Treatment Options

### 2.1 Types, General Symptoms, and Stages

**FIGURE 1:**
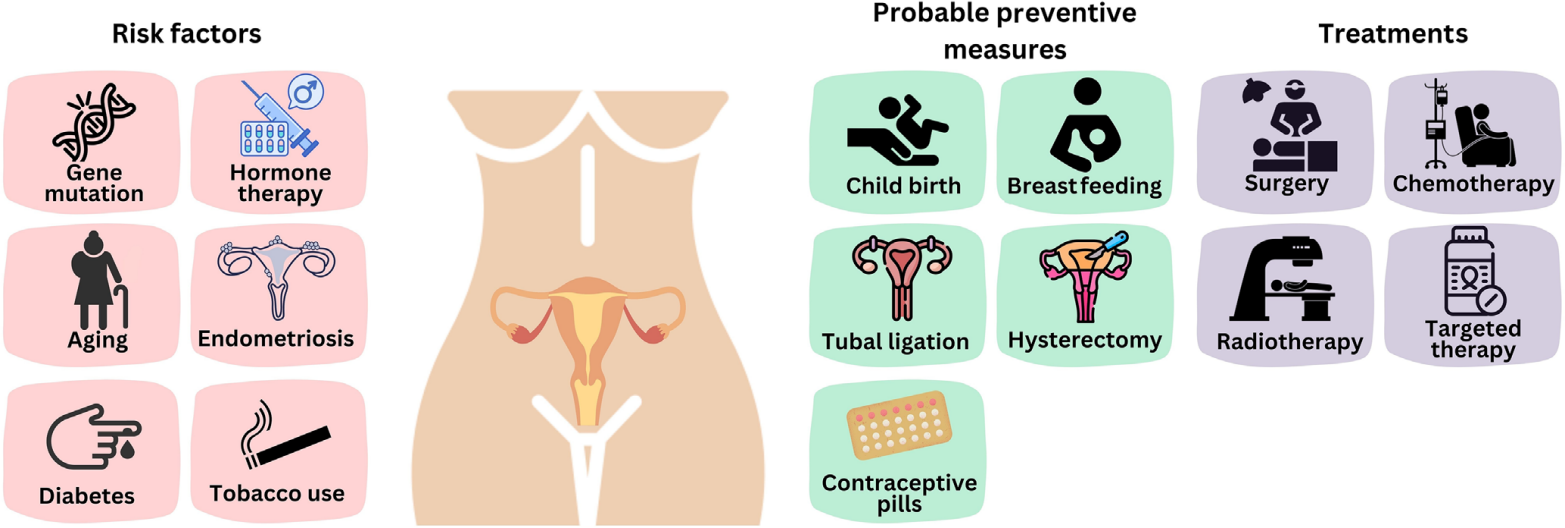
Overview of ovarian cancer: risk factors, prevention, and treatment options.

**FIGURE 2:**
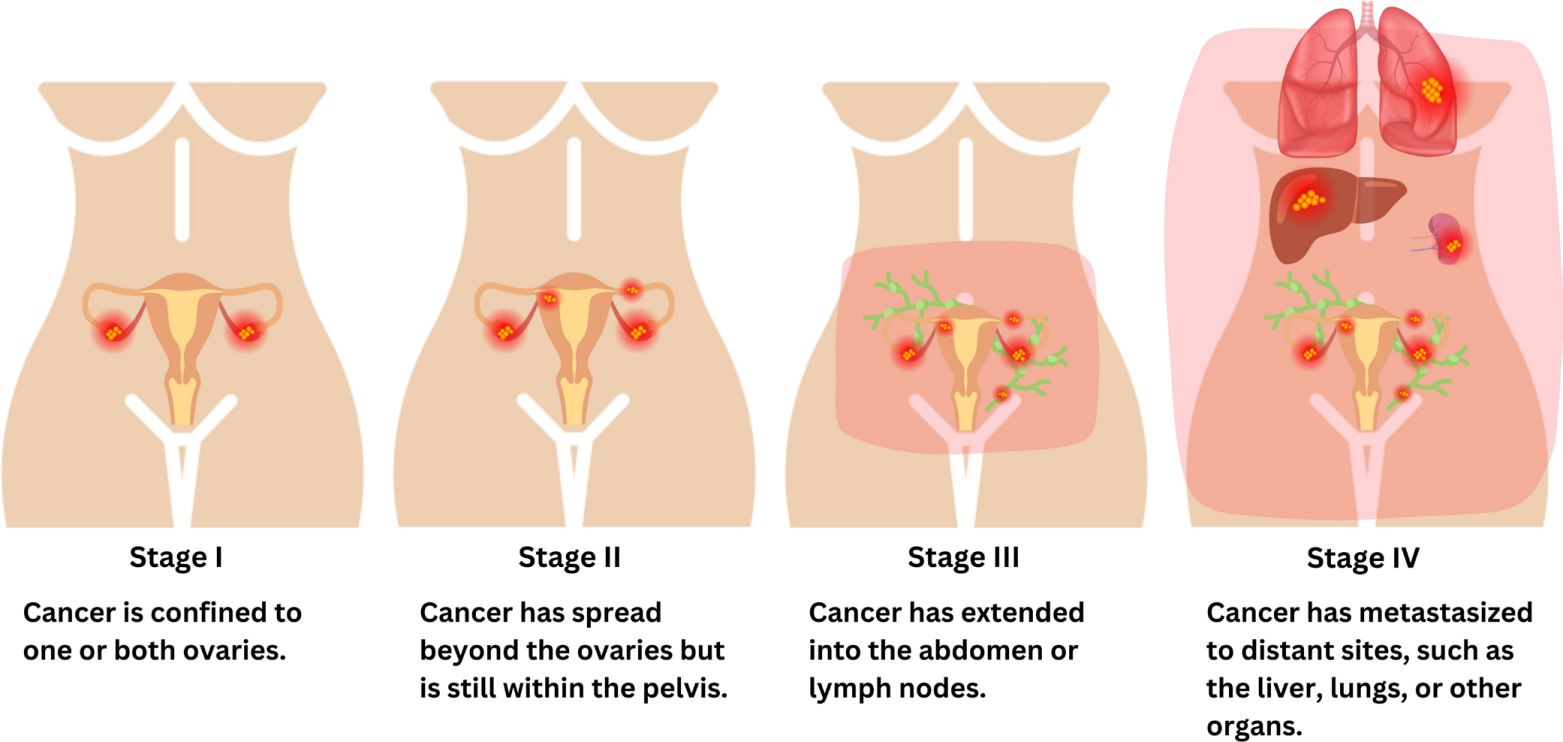
Ovarian cancer is classified into different stages, typically ranging from stage I (early) to stage IV (advanced), based on the extent of the disease.

Ovarian cancers are categorized into three main types: epithelial ovarian carcinomas, germ cell tumors, and stromal cell tumors. Epithelial ovarian carcinomas—accounting for approximately 85%–90% of all cases, originate from the ovarian surface cells. This category includes several histological subtypes, such as serous, endometrioid, clear cell, mucinous, and undifferentiated variants. Germ cell tumors, which account for less than 2% of ovarian cancers, primarily affect younger individuals, and are associated with a 5‐year survival rate of approximately 90%. Stromal cell tumors, comprising about 1% of cases, originate in the ovarian supportive tissues. They are often detected at an early stage and commonly present with symptoms such as abnormal vaginal bleeding (Gaona‐Luviano et al. 2020). Detecting early‐stage ovarian cancer remains a challenge due to its subtle, nonspecific symptoms, which include abdominal bloating, rapid satiety, and increased urination (Orr and Edwards 2018). The risk of ovarian cancer increases with age, particularly in individuals over the age of 50. Those with a history of breast cancer—especially when diagnosed at a younger age—exhibit an elevated risk. Additional risk factors include hormone replacement therapy, tobacco use, asbestos exposure, endometriosis, diabetes, and obesity (Rooth 2013; La Vecchia 2017). While no foolproof preventive measure exists, factors such as the use of oral contraceptives, childbirth, breastfeeding, and certain surgical procedures—including tubal ligation or hysterectomy—may reduce the risk of ovarian cancer (Figure 1) (Lancaster et al. 2015). Ovarian cancer is staged from stage I (early) to stage IV (advanced), based on the extent of disease progression. The stages of ovarian cancer are defined as follows (Menon et al. 2018): Stage I: The cancer is limited to one or both ovaries. Stage II: The cancer has spread beyond the ovaries but remains limited to the pelvic region. Stage III: The cancer has extended to the abdominal cavity and/or lymph nodes. Stage IV: The cancer has metastasized to distant organs, such as the liver, lungs, or other parts of the body (Figure 2).

### 2.2 Mechanisms, Pathophysiology, and Molecular Alterations

Unraveling the complexities of ovarian cancer depends on understanding its intricate molecular landscape, highlighting the pivotal role of genetic alterations in both tumor initiation and progression—insights that are essential for the development of targeted therapies. Notably, mutations in the breast cancer susceptibility genes BRCA1 and BRCA2 significantly increase the risk of ovarian cancer and are implicated in approximately 5%–15% of cases (Kuchenbaecker et al. 2017). Mutations in tumor protein 53 (TP53) contribute to genomic instability and resistance to chemotherapy, particularly in high‐grade serous ovarian cancer (HGSC) (Silwal‐Pandit et al. 2017). In contrast, homologous recombination deficiency (HRD), also prevalent in HGSC, associated with increased sensitivity to poly (ADP‐ribose) polymerase (PARP) inhibitors—an important therapeutic approach (Vergote et al. 2022). Conversely, low‐grade serous ovarian cancer (LGSC) is characterized by mutations in the mitogen‐activated protein kinase (MAPK) pathway, including KRAS and BRAF, distinguishing it from its high‐grade counterpart (Hendrikse et al. 2023). The heterogeneity in estrogen and progesterone expression across ovarian cancer subtypes accentuates the need for tailored hormonal therapies (Modugno et al. 2012). Additionally, emerging insights into microsatellite instability (MSI), deficient mismatch repair (dMMR), tumor mutational burden (TMB), and the diverse subtypes highlight the dynamic and evolving landscape of ovarian cancer research (Roudko et al. 2021). Importantly, these developments highlight the critical role of molecular analysis in guiding clinical decision‐making and informing personalized treatment strategies. The integration of molecular markers into clinical trials reflects a shift toward precision medicine, offering promising advancements in ovarian cancer research and therapeutic outcomes.

Ovarian cancer presents a substantial challenge in the field of oncology, largely due to the development of resistance to conventional chemotherapeutic agents, particularly with cisplatin. Addressing this formidable obstacle requires multifaceted strategies, including the extension of platinum‐free intervals (Sambasivan 2022; Gilbert et al. 2023), the incorporation of non‐platinum cytotoxic agents (Tomao et al. 2017; Dockery et al. 2019), and the advancement of molecularly targeted therapies such as PARP inhibitors (Wu, Xu, et al. 2023), and immunotherapies (Siminiak et al. 2022). Molecular profiling—which includes the evaluation of TMB (Wang et al. 2022), and programmed cell death protein 1 (PD‐1)/programmed death‐ligand 1 (PD‐L1) expression (Dumitru et al. 2022)—significantly enhances the precision of both diagnosis and treatment. Recent advancements in drug delivery systems, particularly those employing nanoparticle‐based strategies, have shown considerable promise in improving therapeutic efficacy (Beyene et al. 2021; Wu, Yang, et al. 2023). However, persistent challenges—such as late‐stage diagnoses, aggressive metastasis, and healthcare disparities—continue to hinder progress in ovarian cancer management. Advancing the field requires a sustained commitment to ongoing research, adequate funding, and cross‐disciplinary collaboration to address the multifaceted nature of the disease. Promising breakthroughs may lie in the strategic integration of existing therapies, the development of more effective treatment protocols, and the cultivation of collaborative frameworks aimed at optimizing patient outcomes.

### 2.3 The Existing Treatment Options

Diagnostic tests for ovarian cancer have traditionally included the CA125 blood test—a widely recognized tumor marker for ovarian cancer and transvaginal ultrasound (Trinidad et al. 2020). However, both methods have notable limitations in sensitivity and specificity. Definitive diagnosis often requires surgical intervention to obtain ovarian tissue for histopathological evaluation. In addition, genetic testing can detect mutations linked with an elevated risk of cancer.

The classification of ovarian cancer staging follows the International Federation of Gynecology and Obstetrics (FIGO) staging system, which ranges from stage 1 to stage 4, and reflects the extent of disease spread. Treatment requires a multidisciplinary approach, combining surgery—ranging from cytoreductive to fertility‐sparing procedures—with chemotherapy using agents like carboplatin and paclitaxel, tailored to tumor pathology and patient health, and administered postoperatively or as standalone therapy in defined cycles. In advanced cases, personalized treatment strategies may include radiotherapy and targeted agents such as olaparib and bevacizumab (Grunewald and Ledermann 2017). Ovarian cancer follow‐up includes clinical assessments and CA125 monitoring. Current treatments face challenges like side effects and chemoresistance (Li et al. 2018). Novel, personalized therapies based on genetic and physiological profiles are crucial for improving outcomes, reducing toxicity, and overcoming treatment resistance.

## 3 Curcumin's Multifaceted Activity in Ovarian Cancer Prevention and Treatment

Curcumin shows therapeutic promise against chronic diseases like cancer, inflammation, and metabolic disorders. It enhances chemotherapy effectiveness, sensitizes cancer cells, and inhibits tumor growth by targeting multiple molecular pathways, making it a major focus in cancer research. Curcumin's interaction with ovarian cancer progression is polygonal, targeting many cellular pathways involved in malignancy. Firstly, it modulates proliferation pathways such as epidermal growth factor receptor (EGFR) (Choe et al. 2018), and activator protein 1 (AP‐1) (Zheng et al. 2022), thereby inhibiting uncontrolled cell growth. Secondly, curcumin disrupts cell cycle progression by downregulating cyclin D1 and cyclin E (Weir et al. 2007; Montopoli et al. 2009), leading to cell cycle arrest and the inhibition of further tumor growth. Additionally, its anti‐inflammatory properties inhibit nuclear factor‐kappa B (NF‐κB) (Fogoros et al. 2006), tumor necrosis factor (TNF) and interleukin‐6 (IL‐6) (Sandhiutami, Arozal, Louisa, and Rahmat 2021), and cyclooxygenase‐2 (COX‐2) (Afshari et al. 2024), suppressing inflammatory responses associated with cancer progression.

Curcumin induces apoptosis in ovarian cancer cells by modulating multiple molecular pathways. It downregulates anti‐apoptotic proteins like BCL‐2, Bcl‐XL, and pro‐caspase‐3, while upregulating pro‐apoptotic markers such as p53 and Bcl‐2‐associated X protein (Bax) (Zheng et al. 2004; Wahl et al. 2007; Seo et al. 2016; Shi et al. 2006; Watson et al. 2010; Ju et al. 2018). Curcumin inhibits survival pathways like the phosphatidylinositol 3‐kinase/protein kinase B (PI3K/Akt) pathway (Alharbi et al. 2024), thereby impeding cancer cell survival and proliferation. In SKOV3 and A2780 cells, curcumin inhibits the PI3K/AKT pathway by decreasing the p‐AKT/AKT ratio in a dose‐dependent manner, enhancing cytotoxicity (Zheng et al. 2006; Yu et al. 2016; Dan et al. 2021). Interestingly, in SKOV3 cells, BCL‐2 activity decreases, but caspase‐3 levels remain unchanged, suggesting a caspase‐3‐independent apoptotic mechanism (Zhao et al. 2017). These findings highlight curcumin's potential to induce apoptosis via cell line‐specific pathways, making it a promising agent for OC treatment. Furthermore, curcumin downregulates anti‐apoptotic proteins like Survivin and BCL‐2, activates p38 MAPK, and triggers apoptosis independently of p53 in SKOV3 cells (Watson et al. 2010; Qian et al. 2011; Chen et al. 2013). In CaOV3 cells, curcumin activates AMPK and induces p53 phosphorylation via p38; this effect decreases with AMPK/p38 inhibition (Pan et al. 2008). When combined with TRAIL, curcumin enhances apoptosis by activating mitochondrial and death receptor pathways (Wahl et al. 2007). Curcumin derivatives like ST03, ST08, and B19 further induce cytotoxicity by activating caspase‐9 and caspase‐3, highlighting their potential as effective agents against OC (Zhang et al. 2012; Qu et al. 2013; Koroth et al. 2019).

**FIGURE 3:**
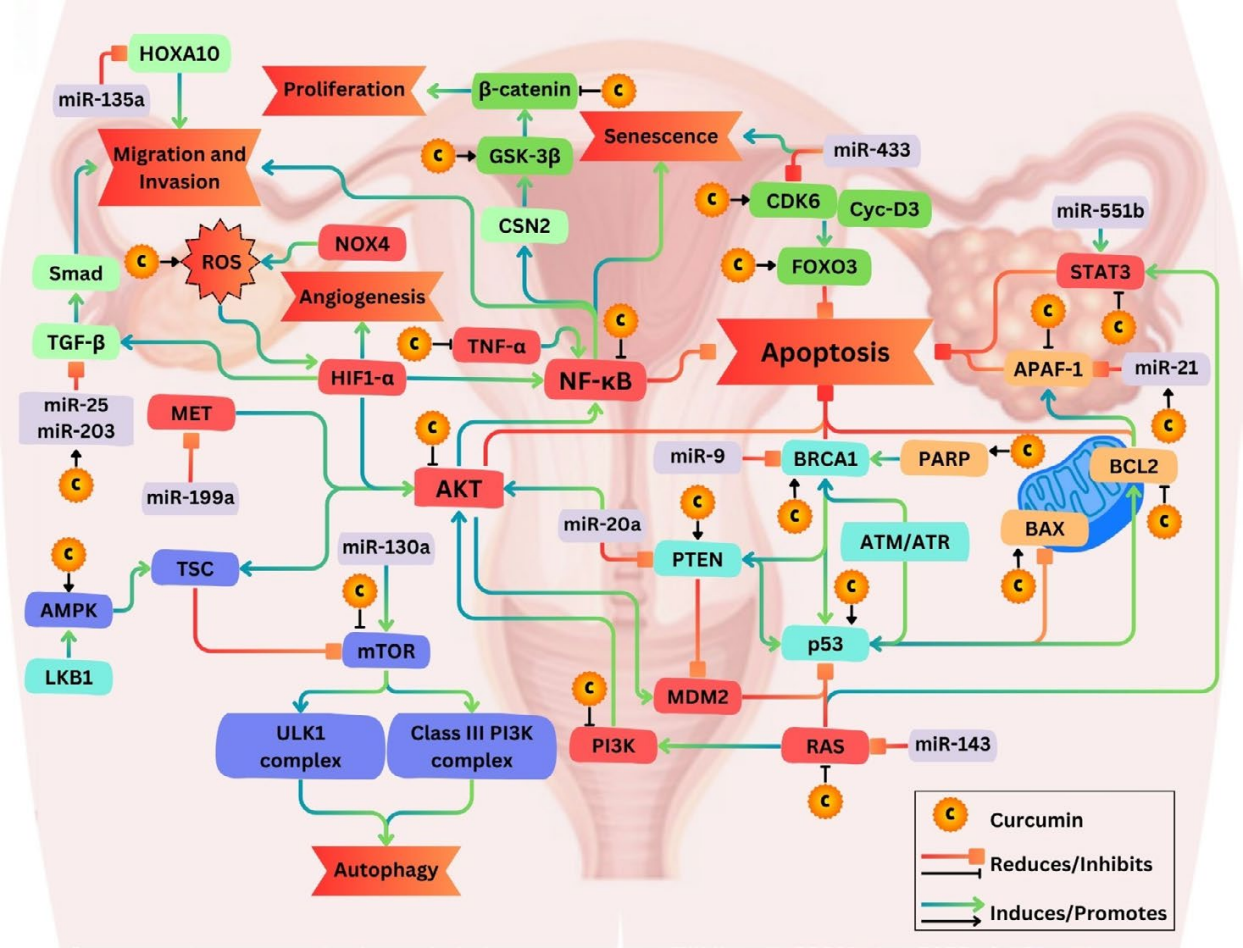
Schematic overview of curcumin's anticancer mechanism in ovarian cancer pathways. Curcumin's diverse anticancer effects in ovarian cancer involve modulation of oncogenes (red boxes: AKT (protein kinase B), RAS (rat sarcoma), NOX4 (NADPH oxidase 4), NF‐κB (nuclear factor‐kappa B), MET (mesenchymal–epithelial transition), HIF1‐α (hypoxia‐inducible factor 1‐alpha), PI3K (phosphoinositide 3‐kinase), MDM2 (mouse double minute 2 homolog), STAT3 (signal transducer and activator of transcription 3), TNF‐α (tumor necrosis factor‐alpha)), tumor suppressors (light blue boxes: p53 (tumor protein 53), PTEN (phosphatase and tensin homolog), LKB1 (liver kinase B1), ATM/ATR (ataxia telangiectasia mutated/ATM and Rad3‐related), BRCA1 (breast cancer 1)), miRNA regulation (violet boxes: miR‐135a, miR‐143, miR‐199a, miR‐20a, miR‐203, miR‐21, miR‐25, miR‐433, miR‐551b), cell cycle regulation (green boxes: CDK6 (cyclin‐dependent kinase 6), β‐catenin (beta‐catenin), GSK‐3β (glycogen synthase kinase 3 beta), FOXO3 (forkhead box O3), Cyc‐D3 (cyclin D3)), apoptosis modulation (light brown boxes: PARP (poly ADP‐ribose polymerase), BCL2 (B‐cell lymphoma 2), BAX (BCL2‐associated X protein), APAF‐1 (apoptotic peptidase activating factor 1)), metabolism/energy sensing mechanisms (blue boxes: AMPK (AMP‐activated protein kinase), TSC (tuberous sclerosis complex), mTOR (mechanistic target of rapamycin), ULK1 complex (Unc‐51 like autophagy activating kinase 1 complex), class III PI3K complex), and other genes relevant to ovarian cancer (light green boxes: TGF‐β (transforming growth factor‐beta), HOXA10 (homeobox A10), CSN2 (casein kinase II subunit alpha), and Smad (mothers against decapentaplegic homolog)).

Furthermore, curcumin suppresses angiogenesis by downregulating vascular endothelial growth factor (VEGF) (Lin et al. 2007), thereby limiting the formation of new blood vessels crucial for tumor growth and metastasis. It also interferes with invasion and metastasis by downregulating matrix metalloproteinase‐9 (MMP‐9) (Pei et al. 2016), and adhesion molecules like integrins, hindering cancer cell migration and invasion into surrounding tissues. Curcumin also interacts with non‐coding RNAs, particularly circular RNAs (circRNAs), which are increasingly recognized as therapeutic targets in ovarian cancer (Ravindran et al. 2023). Emerging evidence highlights its potential to regulate circular RNA networks, notably the circular RNA derived from pleckstrin homology domain‐containing family M member 3/microRNA‐320a/suppressor of morphogenesis in genitalia‐1 (circ‐PLEKHM3/miR‐320a/SMG1) axis, suppressing cell proliferation, inducing apoptosis, and reducing tumorigenesis (Sun and Fang 2021). Curcumin regulates microRNAs (miRNAs), such as miR‐214 (Zhang et al. 2017), miR‐124 (Zhao et al. 2017), and miR‐9 (Liu et al. 2023), affecting cisplatin‐resistance and cell growth. It also upregulates the expression of the tumor‐suppressive long non‐coding RNA (lncRNA), maternally expressed 3 gene (MEG3) (Zhang et al. 2017). Its epigenetic influence, potential in innovative combination therapies, and the challenges associated with its application underscore the need for continued research to unlock the potential of curcumin in revolutionizing ovarian cancer treatment. Schematic overview of curcumin's anticancer mechanism in ovarian cancer pathways is presented in Figure 3.

## 4 Curcumin Nanoformulations for Ovarian Cancer

Despite curcumin's promising therapeutic activities, oral administration of curcumin faces significant challenges due to low water solubility, poor biodistribution and rapid metabolism (Karthikeyan et al. 2020). Clinical studies have shown that only minimal quantities of curcumin are found in the bloodstream, primarily due to extensive metabolic processes in the liver and gut (Cas and Ghidoni 2019). To address these limitations, researchers have developed various curcumin nanoformulations (Hafez Ghoran et al. 2022; Mundekkad and Cho 2023). They have consistently exhibited superior bioavailability, cellular uptake, and anticancer efficacy relative to free curcumin. These advantages are largely attributed to its enhanced physicochemical properties, including improved solubility and stability (Flora et al. 2013; Rahimi et al. 2016; Beyene et al. 2021). Recently, nanocurcumin has emerged as a promising therapeutic strategy in cancer treatment, offering the potential for enhanced efficacy and reduced adverse effects compared to conventional cancer treatments. For instance, compared to free curcumin, curcumin embedded in nanostructured lipid carriers retains its anticancer activity while more effectively reducing cell colony survival, enhancing antitumor efficacy, and improving bioavailability. Preliminary clinical evidence suggests that it may improve patients' quality of life by alleviating treatment‐associated toxicities and enhancing overall therapeutic outcomes (Boroughani et al. 2024). To date, many studies have focused on developing curcumin nanoformulations (i.e., Polymeric, lipid‐based and inorganic) to improve its therapeutic index in ovarian cancer (Mohamadian et al. 2022). Curcumin PLGA nanoparticles have improved the therapeutic efficacy of curcumin in the treatment of ovarian cancer (Yallapu et al. 2010). Bondì et al. (2017) showed that bioavailability of curcumin increased by loading into nano lipid carriers (NLCs) and also NLCs loaded with curcumin have potential drug delivery profiles in the treatment of ovarian cancer treatment and have better anticancer effect than free curcumin. Folic acid‐conjugated nanomicelles encapsulating styrene maleic acid (SMA) and curcumin‐difluorinated (CDF) exhibited potent anticancer effects in ovarian cancer cells by upregulating PTEN expression and inhibiting NF‐κB signaling (Luong et al. 2017). Liu et al. (2024) demonstrated that curcumin nanoparticles significantly enhanced intracellular uptake and effectively suppressed the proliferation and migration of ovarian cancer cells. Chattaraj et al. (2025) developed a novel pH‐responsive and actively targeted formulation for curcumin delivery by combining carboxylated multiwalled carbon nanotubes with folic acid. This formulation enhanced cytotoxicity against SKOV3 cells, while also improving cellular uptake and bioavailability.

## 5 Curcumin on Ovarian Cancer; Insights From Invitro Studies

Over the past two decades, in vitro studies on ovarian cancer cells confirm curcumin's anticancer and pro‐apoptotic effects. Combined with chemotherapy or radiation, it boosts cytotoxicity by inducing apoptosis through specific molecular pathways and well‐defined cancer cell models. Shi et al. (2006) demonstrated that curcumin inhibits growth and promotes apoptosis in Ho‐8910 cells. Treatment with 40 μM curcumin reduced the expression of anti‐apoptotic proteins Bcl‐2 and Bcl‐xL, aswellas with pro‐caspase‐3, while elevating the expression of the Tp53 and the pro‐apoptotic protein Bax. These findings suggest disruption of proliferation and apoptosis pathways, potentially mediated by p53 upregulation. Activation of effector caspase‐3 further supported curcumin's role in apoptosis induction, emphasizing its therapeutic potential in ovarian cancer treatment. Similarly, in A2780 cells, curcumin treatment resulted in growth inhibition and apoptosis, evidenced by the appearance of sub‐G1 peaks, apoptotic morphological changes, decreased NF‐κB expression, and increased caspase‐3 activity (Zheng et al. 2006).

Curcumin has also been shown to inhibit lysophosphatidic acid (LPA)‐induced signaling in SKOV3 ovarian cancer cells, suppressing the generation of reactive oxygen species (ROS), phosphorylation of Akt and extracellular signal‐regulated kinase (ERK), and activation of NF‐κB. These effects resulted in reduced cell proliferation, decreased transcription survival‐related genes, and increased apoptosis, highlighting curcumin's potential as a therapeutic agent targeting ROS‐dependent pathways in ovarian cancer treatment (Saunders et al. 2010). Similarly, Seo et al. (2010) investigated curcumin's inhibitory effects on LPA‐induced motility in the PA‐1 and OVCAR‐3 cell lines. They found that curcumin inhibited the secretion of IL‐6 and interleukin‐8 (IL‐8), phosphorylation of signal transducer and activator of transcription 3 (STAT3), and the co‐immunoprecipitation of phospho‐STAT3 with phospho‐focal adhesion kinase 397 (FAK^397^), thereby impeding cancer cell motility. Several studies have investigated strategies to enhance curcumin's anticancer activity through combination therapy. Treatment with curcumin, either alone or combination with chemotherapy drugs, significantly increased growth inhibition and apoptotic rates in COC1/cisplatin (DDP) cells. Notably, co‐administration of curcumin with chemotherapy resulted in higher inhibition and apoptosis compared to chemotherapy drugs alone, potentially mediated by downregulation of phosphatidylinositol‐4,5‐bisphosphate 3‐kinase catalytic subunit alpha (PI3KCA) expression (Lin et al. 2012).

Arzuman et al. (2014) demonstrated the synergistic effects of curcumin in combination with the monofunctional platinum (II) complex tris (benzimidazole) chloroplatinum (II) (LH4) on human ovarian tumor cells. The study particularly targeted the A2780 cell line and its two variants—one resistant to the chemotherapy drug cisplatin and the other to the cytotoxic platinum agent, ZD0473. Curcumin, administered at varying concentrations and treatment durations, exhibited potent anticancer activity, particularly when pre‐treated or co‐treated with LH4. Notably, increased platinum‐DNA binding was observed, indicating curcumin's potential role as a chemosensitizer in platinum‐based anticancer therapies, and its utility in overcoming drug resistance. A study by Xu et al. (2016) used a novel niosome‐based system for curcumin delivery, which exhibited controlled release and enhanced therapeutic efficacy against ovarian cancer A2780 cells. This study suggested niosomes as a promising strategy for curcumin delivery in cancer therapy. According to Seo et al. (2016) curcumin disrupts calcium homeostasis in OVCAR3 ovarian cancer cells, leading to apoptosis specifically in cancerous cells. This targeted mechanism underscores curcumin's potential as a selective anticancer agent with minimal cytotoxicity to normal cells. Liu et al. (2018) developed a drug delivery system combining curcumin with triptolide, demonstrating significant anticancer activity against SKOV‐3 tumor cells.

To address drug resistance in ovarian cancer, Guo et al. (2020) developed a co‐delivery system designed to enhance the sensitivity of drug‐resistant ovarian cancer cells to cisplatin, with a particular focus on curcumin's modulatory role. Their system significantly increased the cisplatin sensitivity of SKOV3‐DDP cells, indicating its potential to overcome resistance and improve therapeutic outcomes. Similarly, Muhanmode et al. (2022) investigated the combined effects of curcumin, resveratrol, and cisplatin on A2780 cells, demonstrating substantial inhibition of the PI3K/Akt/mTOR signaling pathway. This combination not only reversed cisplatin‐induced pathway activation but also reduced chemoresistance. Curcumin further sensitized ovarian cancer cells to cisplatin, highlighting its promise as part of a therapeutic strategy against chemoresistant disease. Liu et al. (2023) explored curcumin's potential to enhance paclitaxel efficacy in ovarian cancer, showing that co‐treatment significantly inhibited proliferation and induced apoptosis in SKOV3 and A2780 cell lines. These effects were associated with miR‐9‐5p downregulation and BRCA1 upregulation. Huang et al. (2023) identified another mechanism by which curcumin overcomes paclitaxel resistance, demonstrating that it downregulates NF‐κB pathway‐related genes while upregulating smad nuclear‐interacting protein 1 (SNIP1). This upregulation led to reduced expression of pro‐survival genes Bcl‐2 and myeloid cell leukemia 1 (Mcl‐1). Mechanistically, SNIP1 mediated curcumin's inhibitory effect on NF‐κB signaling by suppressing p65 acetylation and promoting its degradation via the EGR1/SNIP1 axis. Additionally, Nourbakhsh et al. (2023) studied the synergistic effects of curcumin and 1‐methyl‐4‐phenylpyridinium (MPP), a chalcone derivative, on inflammatory pathways in breast and ovarian cancer cell lines. Curcumin enhanced the anti‐inflammatory efficacy of MPP by suppressing cytokine production and nitric oxide levels, while increasing inhibitor of kappa B (IκB) concentrations, suggesting its potential as an adjunctive anti‐inflammatory agent in cancer therapy. Recently, Ravindran et al. (2023) demonstrated that curcumin exerts cytotoxic effects in ovarian cancer cells by modulating epigenetic pathways. It alters miRNA expression, inhibits EMT, regulates cancer stem cell markers and DNA repair pathways, modulates ERK/PI3K/Akt signaling, and targets the miR‐199a‐5p/discoidin domain receptor 1 (DDR1) axis to reduce collagen deposition.

Due to its inherently low bioavailability, efforts have been made to enhance the solubility and therapeutic efficacy of curcumin. Yallapu et al. (2010) demonstrated that pretreatment with curcumin sensitized cisplatin‐resistant A2780CP ovarian cancer cells, resulting in reduced required drug doses, downregulation of Bcl‐xL, Mcl‐1, and β‐catenin expression, and an increase in apoptotic markers. Moreover, the authors introduced a nanocurcumin formulation that significantly enhanced curcumin's therapeutic potential, underscoring its promise in ovarian cancer treatment. Similarly, Liu et al. (2016) investigated the co‐administration of paclitaxel and curcumin, finding that curcumin nanoparticles reduced P‐glycoprotein activity, thereby increasing intracellular paclitaxel concentrations and enhancing antitumor efficacy. These findings highlight the critical role of novel drug delivery systems in overcoming multidrug‐resistance and improving chemotherapy outcomes. In another study, Gawde et al. (2018) evaluated the cytotoxic effects of CDF combined with paclitaxel (PTX) on ovarian and cervical cancer cells. Their results showed that folic acid‐decorated nanoparticles containing both CDF and PTX exhibited enhanced cytotoxicity, particularly in folate receptor‐overexpressing cell lines, suggesting promise for targeted combination therapy in gynecological malignancies. Furthermore, Seyed Hosseini et al. (2019) reported that dendrosomal nanocurcumin (DNC), especially when combined with oxaliplatin (Oxa), effectively induced apoptosis in SKOV3 and OVCAR3 ovarian carcinoma cell lines by modulating MMP‐9 expression and inhibiting key metastatic pathways. The formulation also influenced the expression of lncRNAs associated with tumor progression. Zhao et al. (2019) examined the co‐delivery of curcumin and paclitaxel via nanoparticles in SKOV3 cells and their multidrug‐resistant variants. Their findings indicated significant inhibition of cell proliferation and invasion, particularly in drug‐resistant cells, reinforcing curcumin's potential as a complementary therapeutic agent. A comprehensive summary of invitro study findings is presented in Tables 1 and 2.

**TABLE 1:** The summary of in vitro studies investigated the effects and molecular targets of curcumin on ovarian cancer.

S. no.	Cell lines	Dose and duration	Curcumin's effects and molecular targets	References
1	Ho‐8910	10–40 μM, 24 and 48 h	The expression of apoptotic proteins (pro‐caspase‐3, p53, and Bax) increased, while the expression of anti‐apoptotic proteins (Bcl‐2 and Bcl‐XL) decreased	Shi et al. (2006)
2	A2780	10–50 μmol/L, 6 and 24 h	Increased expression of caspase‐3 resulting a suppression of tumor growth	Zheng et al. (2006)
3	A2780CP (Cisplatin‐resistance)	2.5–40 μM, 6 and 48 h	The decrease in β‐catenin, Bcl‐XL, and Mcl‐1 in cisplatin‐resistant cells enhances their sensitivity to chemotherapy	Yallapu et al. (2010)
4	OVCAR3	0–25 μM, 24 h	Anti‐inflammatory effects are attained through the inhibition of NF‐κB, TNF‐α, IL‐6, and IL‐8	Seo et al. (2010)
5	SKOV3	1, 5, 10, and 20 μM, 6–72 h	The suppression of NF‐κB activity through antioxidant properties leads to reduced cell proliferation	Saunders et al. (2010)
6	COC1/DDP (Cisplatin‐resistance)	Various concentrations, 48 h	Elevated curcumin concentrations enhance growth inhibition and apoptosis. Its synergistic effects with cisplatin and paclitaxel further amplify these outcomes. The downregulation of PI3KCA mRNA suggests a potential mechanism underlying the increased apoptosis	Lin et al. (2012)
7	A2780, A2780^cisR^ (Cisplatin‐resistance), and A2780^ZD0473R^ (ZD0473‐resistance)	1.1–17.6, 1.34–21.41, and 1.35–21.52 μM, 72 h	LH4 synergizes in A2780 cells regardless of treatment sequence, with pretreatment enhancing platinum sensitivity, particularly in A2780cisR. Chemosensitization occurs through the reduction of oxidative stress, increased Pt‐DNA binding in A2780 cells, and modulation of NF‐κB, FA/BRCA pathways, and apoptosis	Arzuman et al. (2014)
8	MDAH 2774, SKOV3, and PA1	0, 3, 10, 30, and 90 μM, 24 and 48 h	Apoptosis induction in cells is both concentration‐ and time‐dependent, correlating with increased cytosolic Ca2+ levels. Inhibition of SERCA activity induces cytosolic Ca2+ flux without affecting protein expression	Seo et al. (2016)
9	SKOV3	5–160 μM (Demethoxycurcumin), 12, 24, 36, and 48 h	Curcumin inhibits cell proliferation, induces apoptosis, inactivates the IRS2/PI3K/Akt pathway, and upregulates miR‐551a	Du and Sha (2017)
10	SKOV3, and SKOV3‐TR30 (Multidrug‐ resistance)	0, 10, 20, and 40 μM, 24, 48, and 72 h	Curcumin reduces cell viability, induces apoptotic cell death, triggers protective autophagy, enhances apoptosis by inhibiting autophagy, and directly associates with the AKT/mTOR/p70S6K pathway	Zhao et al. (2019)
11	SKOV3	10 μM, 6 h	Curcumin decreases cell viability and reduces cell adhesion, suppresses fascin expression, impairs cell migration, alters cell morphology and filopodia formation, and inhibits the JAK/STAT3 pathway	Kim et al. (2020)
12	SKOV3	0, 40, and 80 μM, 4 h	Downregulation of p‐AKT, resulting in cytotoxicity	Dan et al. (2021)
13	SKOV‐3, and SKOV3‐DDP (Cisplatin‐resistance)	15 and 100 μM, 24 h	Increased cell death (> 25% of cells with fragmented DNA) is observed, along with a significant accumulation of cells in the G1 and S phases. The expression of PI3K, AKT, and mTOR genes is suppressed, and there is a reduced expression of key antioxidant enzymes and the Nrf2 transcription factor	Hasan et al. (2022)
14	A2780, and A2780^cisR^ (Cisplatin‐resistance)	30 and 70 μM, 48 h	Curcumin, resveratrol, and cisplatin synergistically inhibit the PI3K/AKT/mTOR pathway	Muhanmode et al. (2022)
15	SKOV3, and A2780	SKOV3: 0, 10, 20, 40 μM; A2780: 0, 7.5, 15, and 30 μM, 48 h	Cell viability is reduced, apoptotic cell death is induced, and protective autophagy is activated. Apoptosis is further enhanced by inhibiting autophagy or knocking down LC3B, with a direct connection to the AKT/mTOR/p70S6K pathway	Liu et al. (2023)
16	SKOV3, SKOV3/Txr (Taxol‐resistant), MDAH2774, and 2774/Txr (Taxol‐resistant)	0–60 μM, 72 h	Reduced cell viability and increased apoptosis in taxol‐resistant ovarian cancer cells. SNIP1 upregulation downregulates pro‐survival genes (Bcl‐2 and Mcl‐1), inhibiting NFκB activity through the EGR1/SNIP1 axis	Huang et al. (2023)
17	SKOV3	10 μM, 24 h	Enhanced MPP inhibition reduces NF‐κB DNA binding, suppressing downstream genes such as COX2, MMP‐9, and iNOS. This leads to lower levels of proinflammatory cytokines and NO, while increasing IκB concentration, indicating suppression of the NF‐κB pathway. Additionally, it strengthens the inhibitory effects on NF‐κB DNA binding, proinflammatory cytokines (IL‐1β, IL‐6, TNF‐α), and COX2 gene expression	Nourbakhsh et al. (2023)
18	PA1, and A2780	10–20 μM, 48 h	In PA1 cells, there is downregulation of miR‐335‐5p (targeting ATG5 and OCT4) and miR‐1285 (targeting p53), along with upregulation of miR‐32a (targeting PTEN). In A2780 cells, miR‐181a‐3p (targeting ATG5), miR‐30a‐5p/miR‐216a (targeting BECN1) are upregulated, while miR‐129a‐5p (targeting Bcl‐2) is downregulated. Additionally, inhibition of EMT and reduction of collagen deposition occur via the miR‐199a‐5p/DDR1 axis	Ravindran et al. (2023)
19	SKOV3	10 μM, 24 h	Curcumin augmented inhibition of NF‐κB and STAT3	Afshari et al. (2024)

*Note:* μM, micro molar; μmol/L, micromoles per liter; AKT, protein kinase B; ATG5, autophagy‐related gene 5; Bax, Bcl‐2‐associated X protein; Bcl‐2, B‐cell lymphoma 2; Bcl‐XL, B‐cell lymphoma‐extra‐large; BECN1, beclin 1; Ca2+, calcium ion; COX2, cyclooxygenase‐2; DDR1, discoidin domain receptor tyrosine kinase 1.; EGR1, early growth response 1; EMT, epithelial–mesenchymal transition; FA/BRCA, Fanconi Anemia/breast cancer; G1, gap 1 phase; IL‐1β, interleukin 1 beta; IL‐6, interleukin 6; IL‐8, interleukin 8; iNOS, inducible nitric oxide synthase; IRS2, insulin receptor substrate 2; IκB, inhibitor of nuclear factor‐kappa B; JAK, Janus kinase; LC3B, microtubule‐associated proteins 1A/1B light chain 3B; LH4, LH4 peptide; Mcl‐1, myeloid cell leukemia 1; miR, microRNA; MMP‐9, matrix metalloproteinase‐9; MPP, methyl 6‐(phenylethynyl)‐pyridine; mRNA, messenger ribonucleic acid; mTOR, mammalian target of rapamycin; NF‐κB, nuclear factor‐kappa‐light‐chain‐enhancer of activated B cells; Nrf2, nuclear factor erythroid 2‐related factor 2; OCT4, octamer‐binding transcription factor 4; p53, tumor protein p53; p70S6K, p70 S6 kinase; PA1, prostate cancer cell line; p‐AKT, phosphorylated protein kinase B; PI3K, phosphatidylinositol‐3‐kinase; PI3KCA, phosphatidylinositol‐4,5‐bisphosphate 3‐kinase catalytic subunit alpha isoform; pro‐caspase‐3, pro‐form of caspase‐3; Pt‐DNA, platinum‐DNA; PTEN, phosphatase and tensin homolog; S. no., serial number; S, synthesis phase; SERCA, sarcoplasmic/endoplasmic reticulum calcium ATPase; SNIP1, Smad nuclear‐interacting protein 1; STAT3, signal transducer and activator of transcription 3; TNF‐α, tumor necrosis factor‐alpha.

**TABLE 2:** The summary of in vitro studies investigated the effects and molecular targets of curcumin on ovarian cancer utilizing diverse delivery strategies.

S. no.	Cell lines	Dose and duration	Delivery system	Curcumin's effects and molecular targets	References
1	A2780, and A2780/ADM (Adriamycin‐resistance)	Various concentrations, 24 h	PLGA‐phospholipid nanoparticles containing taxol and curcumin	It overcomes multidrug‐resistance (MDR) in tumor cells, elevates paclitaxel concentration within the cells, and enhances antitumor activity when combined with taxol. Additionally, it reduces P‐gp content and targets mechanisms linked to drug resistance	Liu et al. (2016)
2	A2780	1.5–25 μM, 24 h	Niosome encapsulation of curcumin	The niosome system improves curcumin solubility and cytotoxicity, enhancing cellular uptake, increasing cytotoxicity, inducing cell cycle arrest, and promoting higher apoptosis rates	Xu et al. (2016)
3	SKOV3	6.62 μg/mL	Co‐delivery of triptolide and curcumin via mPEG‐DPPE/CaP nanoparticle	Cell cycle arrest at the S‐phase and G2 transition is induced, leading to apoptosis when combined with triptolide	Liu et al. (2018)
4	SKOV3	250, 500, 750, 1000, and 2000 nM, 72 h	Difluorinated curcumin loaded in albumin nanoparticles	A synergistic anticancer effect is observed when folic acid‐decorated BSA nanoparticles are combined with difluorinated curcumin. This combination enables folate receptor‐mediated targeted uptake and induces apoptosis	Gawde et al. (2018)
5	SKOV3, and OVCAR3	5–55 μM, 24, 48, and 72 h	Oxaliplatin and dendrosomal nanocurcumin	The combination results in higher cell death compared to curcumin alone, with enhanced apoptosis in both cell lines and the greatest overall impact. It also exerts a combined effect on long non‐coding RNA expression in both cell lines	Seyed Hosseini et al. (2019)
6	SKOV3‐DDP (Cisplatin‐resistance)	1–16 μg/mL, 24 and 72 h	Co‐delivery of curcumin and p53 DNA via PEI‐K14 complex	The treatment sensitizes drug‐resistant ovarian cancer cells to DDP, upregulates p53 mRNA, downregulates P‐gp mRNA, and upregulates Bax mRNA	Guo et al. (2020)
7	A2780, and A2780 (3D spheroids)	3.1, 6.2, and 12.5 μg/mL, 24 and 48 h	Co‐delivery of curcumin and cisplatin via lipid‐chitosan hybrid nanoparticles	The combination enhances cytotoxicity compared to curcumin or cisplatin alone. Controlled release behavior contributes to increased efficacy, while curcumin arrests the cell cycle at the G2/M phase. Additionally, it improves penetration into the deeper layers of spheroids	Khan et al. (2020)
8	A2780	0.10 and 0.5 μg/mL, 48 h	Co‐delivery of docetaxel and curcumin via nanomicelles	Docetaxel‐curcumin nanomicelles exhibit stronger inhibition of cell viability compared to curcumin or docetaxel alone, along with increased apoptosis	Hu et al. (2020)
9	SKOV3, and OVCAR3	SKOV3: 10 μM, 24, 48, and 72 h; OVCAR3: 5 μM, 24, 48, and 72 h	Co‐delivery of oxaliplatin and curcumin via dendrosomal nano carrier	Targeting MMPs inhibits ovarian cancer metastasis. Dendrosomal nanocurcumin maximizes cell death and synergizes with oxaliplatin to inhibit growth. Differential expression of MMP‐2 and MMP‐9 was observed	Seyed Hosseini et al. (2023)
10	OVCAR3	2.312 ± 0.27 μg/mL, 24 h	Co‐encapsulation of curcumin and paclitaxel via non‐ionic surfactant‐based nanovesicles	The combination with paclitaxel enhances therapeutic efficacy, resulting in a higher rate of apoptosis. It also downregulates AKT‐1 gene expression and inhibits NF‐κB activity	Haghi Karamallah et al. (2024)

*Note:* μg/mL, microgram per milliliter; μM, micro molar; μM, micro molar; AKT‐1, protein kinase B; Bax, Bcl‐2‐associated X protein; BSA, bovine serum albmin; DDP, cisplatin; G2/M, gap 2/mitosis transition phase; G2, gap 2 phase; MDR, multidrug‐resistance; MMP‐2, matrix metalloproteinase‐2; MMP‐9, matrix metalloproteinase‐9; MMPs, matrix metalloproteinases; mPEG‐DPPE/CaP, methoxy polyethylene glycol‐dipalmitoylphosphatidylethanolamine/calcium phosphate; mRNA, messenger RNA; NF‐kB, nuclear factor‐kappa B; nM, nanomolar; p53, tumor protein p53; PEI‐K14, polyethyleneimine‐the bifunctional peptide K14; P‐gp, P‐glycoprotein; PLGA, poly (lactic‐co‐glycolic acid); S. no., serial number; S‐phase, synthesis phase.

Furthermore, several studies have compared or investigated the synergistic effects of curcumin in combination with other natural compounds. Chan et al. (2003) showed that curcumin and quercetin increased the sensitivity of ovarian cancer cell lines CAOV3 and SKOV3 to cisplatin, both when co‐administered and when applied 24 h prior to cisplatin treatment. Yunos et al. (2011) investigated the sequential application of cisplatin in combination with epigallocatechin‐3‐gallate (EGCG) and curcumin, and reported that lower concentrations and a shorter time interval between the two additions resulted in a greater cytotoxic effect. The novel core–shell nano particles loaded with resveratrol exhibited minimal cytotoxicity in human ovarian cancer cells (MDAH‐2774 and SKOV‐3 cells), whereas the same system loaded with curcumin significantly inhibited mitochondrial activity. The MDAH‐2774 cell line was more sensitive to the treatment than SKOV‐3 (Weżgowiec et al. 2025). Computational analysis revealed strong binding of mangiferin and curcumin to PI3K, Akt, and mTOR. Exosome/liposome‐based delivery enhanced their bioavailability and cellular uptake, suggesting potential as effective agents in ovarian cancer treatment (Alharbi et al. 2024).

## 6 Curcumin on Ovarian Cancer; Perceptions From Invivo Studies

In vivo animal studies have highlighted curcumin's potential in promoting ovarian health and combating disease (Tables 3 and 4). Lin et al. (2007) demonstrated that curcumin, both alone and in combination with docetaxel, significantly inhibited multidrug‐resistant ovarian tumor growth in mice by 58%. Additionally, a curcumin–paclitaxel nanoemulsion was shown to suppress the expression of drug resistance‐associated proteins in SKOV3 tumor‐bearing mice. This combinatorial therapy not only inhibited tumor proliferation, angiogenesis, and growth but also promoted apoptosis in tumor cells, underscoring its potential to improve treatment outcomes in drug‐resistant ovarian cancer. Tang et al. (2010) evaluated the antitumor efficacy of polyacetal‐based polycurcumin in a SKOV3 xenograft mouse model. Mice treated with polycurcumin exhibited a 68% reduction in tumor growth compared to controls, with tumor mass decreasing markedly from 1.57 to 0.49 g, highlighting polycurcumin's potent tumor‐suppressive properties. Ganta et al. (2010) explored the combined effects of curcumin and paclitaxel (PTX) in a nanoemulsion formulation in SKOV3 tumor‐bearing mice. Curcumin pretreatment significantly reduced intestinal P‐glycoprotein (Pgp) and cytochrome P450 3A2 (CYP3A2) levels, enhancing the oral bioavailability of PTX. This combination exhibited enhanced antitumor activity without inducing acute toxicity, suggesting a promising approach for improving ovarian adenocarcinoma therapy. Zhao et al. (2017) reported that the combination of dihydroartemisinin (DHA) and curcumin significantly suppressed tumor growth in a xenograft nude mouse model without apparent toxicity, indicating its therapeutic potential in ovarian cancer.

**TABLE 3:** The effect of curcumin on ovarian cancer in various in vivo models: Insights into dosages, duration, administration routes, molecular targets, and effects.

S. no.	In vivo model	Dose, duration, and route of administration	Curcumin's effects and molecular targets	References
1	Orthotopic murine model	500 mg/kg for 5 weeks, p.o.	The combination with docetaxel reduces tumor growth and drug resistance, while suppressing proliferation and angiogenesis	Lin et al. (2007)
2	Athymic nude mouse xenograft	100 mg/kg (Polyacetal‐based polycurcumin), single dose, i.v.	Reduction in tumor growth.	Tang et al. (2010)
3	Murine model	50 mg/kg for 3 days, p.o.	The improved oral bioavailability of paclitaxel enhances its antitumor activity	Ganta et al. (2010)
4	Nude mouse xenograft	20 mg/kg for 5 weeks, p.o.	The combination with dihydroartemisinin significantly inhibits tumor growth without causing obvious toxicity	Zhao et al. (2017)
5	Hen model	25.8 mg/day, 53.0 mg/day for 12 months, p.o.	The treatment leads to a reduction in ovarian cancer incidence, tumor sizes, and the number of tumors. It inhibits NF‐κB and STAT3 signaling pathways, induces the nuclear factor erythroid 2/heme oxygenase 1 antioxidant pathway, and results in fewer KRAS and HRAS mutations in ovarian tumors	Sahin et al. (2018)
6	Nude mouse xenograft	10 mg/kg for 10 days, p.o.	The combination exhibits synergistic anticancer effects with paclitaxel, reversing paclitaxel resistance and enhancing its antitumor activity	Zhao et al. (2019)
7	Sprague Dawley rats	500 mg/kg (Curcumin or nanocurcumin), single dose, p.o.	The pharmacokinetic parameters are comparable in plasma and organs, except for the ovaries. Nanocurcumin results in higher curcumin levels in plasma, liver, kidney, and colon, with 3.6 times higher curcumin concentrations in the ovaries	Arozal et al. (2019)
8	Xenogeneic nude mouse model	5 mg/kg, daily for 20–25 days, p.o.	The treatment specifically targets the tumor microenvironment in the MC38 murine colon cancer model, promoting the induction of tumor antigen‐specific T cells. It restores the activity of dendritic cells (DCs) in tumor tissues, inhibits STAT3 transcription activity in CD11c + DCs, and modulates the expression of immune‐related markers on DCs, such as CD83 and PD‐L1	Hayakawa et al. (2020)
9	Wistar rats with DMBA‐induced ovarian cancer	100 mg/kg BW (Curcumin or nanocurcumin) daily for 4 weeks, p.o.	The treatment results in a significant reduction in ovarian tumor volume and weight. It downregulates Ki67, TGF‐β, and PI3K, and inhibits Akt phosphorylation. Additionally, there is a reduction in JAK expression, STAT3 phosphorylation, and IL‐6 concentrations. Proliferation is inhibited through the downregulation of the PI3K/Akt and JAK/STAT3 signaling pathways	Sandhiutami, Arozal, Louisa, and Rahmat (2021), Sandhiutami, Arozal, Louisa, Rahmat, and Wuyung (2021)
10	BALB/c athymic mice	15 mg/kg every 2 days for 5 weeks. p.o.	Tumor volume and weight were significantly reduced, with suppression of tumor growth observed at higher circ‐PLEKHM3 levels. The expression of CASP3 and Bax was promoted, while PCNA expression	Sun and Fang (2021)
11	Wistar rats with DMBA‐induced ovarian cancer	100 mg/kg BW (Curcumin) daily for 4 weeks, p.o.	The co‐treatment of curucmin with cisplatin improved markers of mitochondrial biogenesis (PGC‐1α, TFAM) and suppressed ET‐1‐mediated renal fibrosis and apoptosis. It also suppressed ET‐1 expression, activated ETBR, enhanced NRF2 expression, inhibited renal injury, preserved kidney function, and regulated the ET‐1/ETBR/ETAR signaling pathway	Barinda et al. (2022)
12	Wistar rats with DMBA‐induced ovarian cancer	100 mg/kg BW (curcumin or nanocurcumin) daily for 4 weeks, p.o.	Nanocurcumin combined with cisplatin alleviates kidney function markers and hematological abnormalities, leading to decreased plasma urea, creatinine, and NGAL levels. It increases glutathione activities, reduces lipid peroxidation, and decreases plasma TNF‐α. The combination exerts both antioxidant and anti‐inflammatory effects	Louisa et al. (2023)
13	BALB/c nude mice, subcutaneous xenograft	20 mg/kg twice a week for 21–28 days, i. p.	The combination treatment with PTX decreases miR‐9‐5p expression, increases BRCA1 expression, and reduces Ki‐67 levels	Liu et al. (2023)

*Note:* μg/g, micrograms per gram; Akt, protein kinase B; BRCA1, breast cancer type 1 susceptibility protein; BW, body weight; CASP3, caspase‐3; CD11c + DCs, cluster of differentiation 11c positive dendritic cells; CD83, cluster of differentiation 83; DCs, dendritic cells; ET‐1, endothelin‐1; ETAR, endothelin A receptor; ETBR, endothelin B receptor; i.p., intraperitoneal; i.v., intravenous; IL‐6, interleukin‐6; JAK, Janus kinase; kg, kilograms; Ki67, marker of proliferation Ki‐67; KRAS and HRAS, Kirsten and Harvey rat sarcoma viral oncogene homolog; MC38, murine colon carcinoma cell line; mg, milligrams; miR, microRNA; NF‐κb, nuclear factor‐kappa B; NGAL, neutrophil gelatinase‐associated lipocalin; NRF2, nuclear factor erythroid 2‐related factor 2; p.o., per os (referring to oral administration); PCNA, proliferating cell nuclear antigen; PD‐L1, programmed death‐ligand 1; PGC‐1α, peroxisome proliferator‐activated receptor gamma coactivator 1‐alpha; PI3K, phosphoinositide 3‐kinase; PTX, paclitaxel; S. no, serial number; STAT3, signal transducer and activator of transcription 3; TFAM, mitochondrial transcription factor A; TGF‐β, transforming growth factor‐beta; TNF‐α, tumor necrosis factor‐alpha.

**TABLE 4:** The impact of curcumin on ovarian function in various in vivo models: Insights into dosages, duration, administration routes, molecular targets, and effects.

S. no.	In vivo model	Dose, duration, and route of administration	Curcumin's effects and molecular targets	References
1	CBA mice	100 μg/g, i.g.	The treatment improves oocyte development and reduces apoptosis and necrosis in immune‐related tissues	Alekseyeva et al. (2011)
2	Wistar albino rats	200 mg/kg for 2 or 4 h, i.p.	The treatment demonstrates potential protective effects against ovarian ischemia–reperfusion injury	Eser et al. (2015)
3	Kunming mice	0, 100, 150, or 200 mg/kg for 21 days, i.g.	The intervention reduces oxidative stress, increases antioxidant capacity, and decreases the number of atretic follicles	Wang et al. (2017)
4	C57BL/6 female mice	100 mg/kg for 42 days, i.p.	The treatment improves hormonal balance, enhances antioxidant capacity, exhibits anti‐aging effects, and reduces apoptosis in granulosa cells	Yan et al. (2018)
5	Wistar rats	100 mg/kg (free curcumin) and 1 mg/kg (nanocurcumin) for 2.5 or 3 h, i.p.	Superior antioxidant defense and reduced oxidative stress with nanocurcumin	Behroozi‐Lak et al. (2018)
6	Wistar rats	50 mg/kg, 100 mg/kg, 200 mg/kg (nanocurcumin) for 15 days, p.o.	The intervention alleviated oocyte reduction and exhibited anti‐apoptotic effects. It reduced sex hormone disturbances in PCOS, improved oxidative stress markers, and decreased elevated TNF‐α levels. The treatment restored PI3K/AKT/mTOR levels, alleviated insulin resistance, improved β‐cell function, and preserved islet integrity. It also normalized sex hormone levels	Abuelezz et al. (2020)
7	Rat model	200 mg/kg for 2 weeks	The treatment exhibits ovarioprotective effects, downregulates serum testosterone, and restores insulin resistance (IR). It inhibits inflammatory cell infiltration in ovarian tissues, decreases the expression of IRS1, PI3K, and AKT, and increases the expression of GLUT4 and PTEN	Zheng et al. (2022)

*Note:* μg/g, micrograms per gram; GLUT4, glucose transporter 4; i.g., intragastric; i.p., intraperitoneal; IR, insulin resistance; IRS1, insulin receptor substrate 1; kg, kilograms; mg, milligrams; p.o., per os (Referring to oral administration); PCOS, polycystic ovary syndrome; PI3K/AKT/mTOR, phosphoinositide 3‐kinase/protein kinase B/mammalian target of rapamycin; PTEN, phosphatase and tensin Homolog; S. no., serial number; TNF‐α, tumor necrosis factor‐alpha.

Sahin et al. (2018) investigated the effect of daily curcumin intake on spontaneous ovarian cancer in hens. Curcumin supplementation significantly reduced tumor incidence and progression. Mechanistically, curcumin inhibited the NF‐κB and STAT3 pathways, activated antioxidant signaling, and reduced mutations in Ras family oncogenes, supporting its role as a preventive and therapeutic agent in ovarian cancer. Zhao et al. (2019) developed a nanocarrier system for the co‐delivery of curcumin and paclitaxel, which showed significant antitumor efficacy in ovarian tumor‐bearing nude mice, highlighting its promise as an innovative therapeutic strategy. Sandhiutami, Arozal, Louisa, Rahmat, and Wuyung (2021) improved curcumin's bioavailability by encapsulating it in chitosan‐sodium tripolyphosphate nanoparticles. Nanocurcumin achieved a 20‐fold increase in plasma concentration compared to free curcumin. In combination with cisplatin, it synergistically reduced the phosphorylation of PI3K, JAK, STAT3, and Akt, enhancing anticancer efficacy in a rat model. Beyond its anticancer properties, curcumin also shows therapeutic potential in ovarian‐related disorders due to its anti‐inflammatory, antioxidant, and anti‐apoptotic activities. Alekseyeva et al. (2011) demonstrated that in CBA female mice with ovarian immune disorders, intragastric administration of curcumin (100 μg/g, four times weekly) significantly enhanced oocyte maturation (metaphases I and II), reduced apoptosis and necrosis in immune‐affected tissues, and lowered circulating stab neutrophils, suggesting reproductive and immunomodulatory benefits.

In a rat model of ovarian ischemia–reperfusion injury, Eser et al. (2015) found that although 200 mg/kg curcumin did not significantly affect oxidative or histological parameters during ischemia–reperfusion, it resulted in improved ovarian histological scores, indicating potential tissue‐protective effects. Wang et al. (2017) administered curcumin at varying doses (0, 100, 150, 200 mg/kg) for 21 days in female Kunming mice with induced ovarian oxidative stress. Curcumin treatment significantly decreased ROS and malondialdehyde (MDA) levels while increasing superoxide dismutase (SOD) activity, reducing oxidative damage and atretic follicle numbers, and suggesting a dose‐dependent improvement in ovarian health. In a mouse model of premature ovarian failure (POF), Yan et al. (2018) observed that curcumin administration restored hormonal balance by increasing estrogen and progesterone levels and decreasing FSH and LH. It also elevated SOD levels, reduced MDA, and downregulated aging and oxidative stress markers while increasing anti‐Müllerian hormone (AMH) expression. Furthermore, it inhibited granulosa cell apoptosis and promoted anti‐apoptotic and anti‐inflammatory protein expression, indicating potential fertility benefits. Behroozi‐Lak et al. (2018) compared curcumin and nanocurcumin in a rat model of ovarian ischemia–reperfusion injury, reporting that nanocurcumin exerted superior effects by enhancing antioxidant defenses and reducing oxidative stress markers, highlighting the significance of dosage and formulation. In autoimmune ovarian disease models, Abuelezz et al. (2020) found that curcumin alleviated oocyte depletion and demonstrated anti‐apoptotic effects in ovarian tissues. In d‐galactose‐induced POF mice, curcumin suppressed apoptosis‐related protein expression and reduced granulosa cell apoptosis, further supporting its potential as a therapeutic agent in ovarian dysfunction.

## 7 Curcumin on Ovarian Cancer; Current Status of Human Clinical Studies

As of March 2024, the International Clinical Trials Registry Platform (ICTRP) lists a total of 923 studies investigated the therapeutic potential of curcumin, covering the period from 1900 to 2024. A significant proportion of these studies employed double‐blind, randomized, and placebo‐controlled trial designs, and consistently reported favorable effects on both clinical outcomes and relevant biomarkers. Notably, metabolic disorders—particularly those associated with obesity and insulin resistance—represent the most extensively studied category, followed by musculoskeletal disorders (Panknin et al. 2023). Of the registered clinical studies, 155 have been completed; however, only 40 have yielded public available results. Within this body of research, 118 studies have focused on oncology, with 26 completed and 14 providing accessible outcomes. These cancer‐focused trials have investigated curcumin's therapeutic potential across a range of cancers including, breast cancer (Ryan et al. 2013; Miller 2018; Ryan Wolf et al. 2018; Kalluru et al. 2020; Saghatelyan et al. 2020), colorectal cancer (Carroll et al. 2011; Gunther et al. 2017; Cruz‐Correa et al. 2018; Howells et al. 2019), prostate cancer (Suzuki et al. 2006; Hejazi et al. 2013, 2016; Choi et al. 2019; Saadipoor et al. 2019), pancreatic cancer (Dhillon et al. 2008; Parsons et al. 2016).

Despite encouraging preclinical findings on curcumin's effects in ovarian cancer, clinical evidence supporting its efficacy remains limited. Currently, no pilot studies are underway to evaluate the therapeutic effects of curcumin supplementation specifically in ovarian cancer patients. Nevertheless, ovarian cancer shares significant molecular and pathological associations with other malignancies such as breast, colorectal, endometrial, and liver cancers (Zheng et al. 2018). Clinical trials in these related cancers have demonstrated the safety and potential efficacy of curcumin. In breast cancer patients, curcumin has been studied for its impact on a range of clinical outcomes, including radiation‐induced dermatitis, modulation of inflammatory biomarkers, pain management, overall well‐being, and chemotherapy response rates (Ryan et al. 2013; Miller 2018; Ryan Wolf et al. 2018). Similarly, studies in colorectal cancer patients have reported that curcumin is well‐tolerated by healthy tissues (Garcea et al. 2005). Moreover, Bayet‐Robert et al. (2010) investigated the combination of docetaxel and curcumin in 14 patients with metastatic or advanced‐stage breast cancer, indicating the feasibility and potential of such combinatorial approaches in clinical oncology. In this study, six escalating doses of curcumin were evaluated in combination with docetaxel. The maximum tolerated dose was determined to be 6 g/day, while dose‐limiting toxicities (DLTs), primarily grade III diarrhea, emerged at the highest tested dose of 8 g/day. Hematological toxicities were transient and manageable. Notably, significant reductions in carcinoembryonic antigen (CEA) and VEGF levels were observed, indicating potential antiangiogenic activity. Clinically, no cases of disease progression were reported; five patients exhibited partial responses, and three maintained stable disease. These findings highlight the potential efficacy and safety of the curcumin‐docetaxel combination in advanced breast cancer and support its further investigation in a phase II clinical trial. Transitioning curcumin research in ovarian cancer from preclinical to clinical studies presents several major challenges. Curcumin's poor bioavailability mainly restricts its in vivo efficacy. Many preclinical studies rely on high doses or nanoformulations that are not yet standardized for clinical use, complicating translation to human trials. In vitro models often fail to replicate the complexity of the tumor microenvironment, limiting their predictive value. Variations in experimental design, including differences in dosage, treatment duration, and the choice of cell lines or animal models, contribute to inconsistent results. Clinical studies, though few, often suffer from small sample sizes, short durations, and lack of controls. The absence of clearly defined clinical endpoints and validated biomarkers further weakens the strength of these studies. Additionally, potential synergistic effects of curcumin with other therapeutic agents remain unexplored in clinical studies. The biological heterogeneity of ovarian cancer adds another layer of complexity, making uniform treatment responses unlikely. Inconsistent formulation quality and lack of pharmacokinetic monitoring also contribute to mixed results.

## 8 Conclusions and Future Perspectives

Over the past two decades, research has shown that curcumin enhances ovarian cancer treatment by sensitizing cancer cells to chemotherapy and radiotherapy, inhibiting resistance mechanisms, and regulating apoptotic, anti‐proliferative, and anti‐metastatic pathways. In vitro and in vivo studies support its concentration‐dependent tumor‐suppressive effects, including restoring hormonal balance, reducing oxidative stress, and lowering inflammation. This integrated approach offers improved therapeutic efficacy and hope for patients with drug‐resistant or treating advanced stage ovarian cancer. While numerous in vitro and animal studies have demonstrated its anticancer effects, these results often fail to translate effectively to human trials due to biological differences between experimental models and patients. There is also a lack of standardized formulations and dosing strategies, with various studies employing different delivery systems and concentrations, making comparison and replication difficult. Pharmacokinetic data are often incomplete or inconsistent, leading to uncertainty regarding the optimal therapeutic range. Large‐scale studies are needed to validate its effectiveness, evaluate safety over the long term, and guide further research on disease progression, optimal dosing, delivery methods, and patient subgroup identification. Also, rigorous assessment of tumor response, progression‐free survival (PFS), and overall survival (OS) is essential for determining the therapeutic efficacy of curcumin‐based treatments. Patient‐derived organoids have recently emerged as valuable tools in cancer research for drug screening, target identification, and the development of personalized medicine. However, to date, no studies have investigated the effects of curcumin on ovarian cancer organoids. Therefore, future studies should employ organoid models to generate clinically relevant data that may inform the design and implementation of curcumin‐based clinical trials.

Native curcumin exhibits poor bioavailability, necessitating high doses that may cause gastrointestinal disturbances, hepatic stress, or interfere with chemotherapy via drug–drug interactions. To overcome these challenges, various strategies are being investigated to improve its absorption and bioavailability. These include novel oral delivery systems, lipid‐based formulations, and nanostructured carriers. Protecting curcumin from degradation is essential, with promising approaches like metallocomplexation (Prasad et al. 2021) and nanoparticle encapsulation (Feltrin et al. 2022; Mahmoudi et al. 2022; Bashkeran et al. 2023) showing significant potential. Recently, the application of artificial intelligence (AI), particularly machine learning techniques, to optimize curcumin nanoformulations emerged as a prominent area of research. Studies have demonstrated that artificial neural networks (ANN) can improve the biological properties of curcumin and facilitate the design of more efficient drug delivery systems. Therefore, further research in this direction is warranted, as it may yield more robust and translationally relevant outcomes. The successful development of curcumin nanoformulations may complement existing therapies, offering meaningful benefits to patients. However, the successful translation of curcumin nanoformulations into clinical trials requires addressing several major factors, including formulation optimization, dose selection, purity, sourcing, toxicity, and understanding of mechanisms of action. Physicochemical characterization is essential for evaluating the structural and functional properties of these formulations to ensure compliance with clinical translation standards. Although nanoformulations improve the bioavailability and therapeutic efficacy, they may also introduce new risks, including nanoparticle‐induced toxicity, immune responses, and organ accumulation. Moreover, curcumin's antioxidant properties may counteract the pro‐oxidant mechanisms of certain chemotherapeutic agents. These issues contribute to limited and inconsistent evidence of therapeutic benefits in clinical trials (Nelson et al. 2017). Therefore, extensive clinical trials with large patient populations are necessary to validate the safety and efficacy of curcumin and its nanoformulations.

Elucidating curcumin's mechanisms of action holds significant potential for developing tailored therapeutic strategies aligned with the molecular landscape of ovarian cancer. Curcumin has shown to efficacy in inhibiting the Fanconi anemia (FA)/BRCA DNA repair pathway, which is associated with resistance to DNA cross‐linking agents such as cisplatin (DDP). By suppressing the monoubiquitination and nuclear foci formation of Fanconi anemia group D2 protein (FANCD2), curcumin sensitizes cisplatin‐resistant cancer cells to cisplatin treatment, thereby enhancing cytotoxicity and promoting apoptosis (Xiao et al. 2010; Chen et al. 2015). Further investigation into curcumin's ability to inhibit proteasome function—an upstream regulator FA/BRCA pathway activation—may reveal additional mechanisms to overcome cisplatin‐resistance in ovarian cancer. In oral cancer models, the combination of curcumin and the PARP inhibitor olaparib has demonstrated synergistic effects in inducing DNA damage and disrupting DNA repair processes. This combination therapy could similarly enhance the DNA damage accumulation in ovarian cancer cells, ultimately inducing selective cell death (Molla et al. 2021).

Curcumin's ability to exploit synthetic lethality interactions represents a promising strategy for targeting specific vulnerabilities in ovarian cancer cells. Similar to MEK inhibitors, curcumin induces synthetic lethality when combined with Food and Drug Administration (FDA)‐approved targeted anticancer drugs such as regorafenib, particularly in KRAS mutant tumors (Wu et al. 2019). Investigating how curcumin induces autophagy and apoptosis may elucidate its antitumor mechanisms in ovarian cancer and contribute to the refinement of therapeutic strategies. Moreover, the mechanism by which curcumin promotes the ubiquitination and destabilization of mutant p53—as demonstrated in squamous cell carcinoma—may represent a novel targeted therapeutic approach. By rendering mutant p53 signaling inactive, curcumin suppresses its oncogenic activity without disrupting the tumor‐suppressive functions of wild‐type p53 (Oak et al. 2023).

Exploring advanced pharmaceutical strategies, including nanoparticle‐based delivery systems and combination therapies with deubiquitinase (DUB) inhibitors—may enhance curcumin's efficacy against mutant p53‐expressing ovarian cancer cells. Additionally, curcumin's ability to modulate the inflammatory microenvironment,—particularly in the context of varying p53 mutation statuses—presents a promising avenue for immunomodulatory approaches in ovarian cancer treatment. By inhibiting pro‐tumor signaling pathways and reprogramming macrophage polarization, curcumin may suppress tumorigenesis and enhance antitumor immune responses (Xu et al. 2022). Curcumin’ affects key signaling pathways involved in cancer progression, including the wingless‐related integration site (Wnt)/β‐catenin and retinoblastoma protein (Rb)/cyclin‐dependent kinase (CDK)/E2F pathways. Combination therapies involving curcumin with S‐phase inhibitors or cyclin‐dependent kinase 4/6 (CDK4/6) inhibitors may synergistically inhibit ovarian tumor cell proliferation and help overcome mechanisms of drug resistance (Li et al. 2022).

The potential of curcumin as a therapeutic agent for ovarian cancer is increasingly supported by studies demonstrating its ability to modulate epigenetic modifications in ovarian cancer cells (Wu et al. 2020; Ming et al. 2022; Jafari‐Nozad et al. 2023). Specifically, curcumin has been shown to modulate DNA methylation, histone modifications, and microRNA expression, potentially reversing the aberrant gene expression patterns characteristic of ovarian cancer (Xie et al. 2021), and thereby inhibiting tumor growth and metastasis. Curcumin's influence on non‐coding RNAs, including circRNAs and miRNAs, presents a compelling opportunity for therapeutic intervention, potentially modulating a key processes such as cisplatin‐resistance and tumorigenesis (Yang et al. 2020; Li et al. 2021; Sun and Fang 2021; Si et al. 2023). However, significant knowledge gaps remain concerning the direct role of curcumin‐induced epigenetic modifications in mediating its anticancer effects. The mechanisms by which these modifications orchestrate gene expression and regulate major signaling pathways in carcinogenesis remain poorly understood. Furthermore, exploring synergistic interactions with established epigenetic therapies—such as DNA methyltransferase inhibitors (Nowak et al. 2017), and histone deacetylase inhibitors (Shetty et al. 2021; Guo and Wang 2022)—may enhance therapeutic efficacy. Moreover, the use of single‐cell‐based omics approaches to investigate curcumin intervention in ovarian cancer is still awaiting investigation. These approaches offer the potential to elucidate molecular mechanisms underlying curcumin‐mediated regulation at various stages of cancer progression and therapeutic response. Single‐cell analysis also enables target validation and the identification of drug‐sensitive cell populations, facilitating a deeper understanding of resistance mechanisms. This is particularly valuable for characterizing the tumor microenvironment and advancing the clinical application of curcumin‐based antitumor strategies. Eventually, incorporating curcumin into treatment regimens holds significant promise for enhancing therapeutic outcomes while potentially reducing toxicity relative to conventional chemotherapeutic agents. However, realizing curcumin's full therapeutic potential in ovarian cancer will require continued preclinical research and rigorously designed clinical trials. Future research should focus on leveraging multi‐omics approaches, improving drug delivery systems, and conducting rigorous clinical studies to elucidate curcumin's mechanisms of action and assess its safety in humans. Addressing curcumin's bioavailability and stability challenges remains essential for its successful clinical translation in ovarian cancer therapy.

## Author Contributions

**Smirnova Elena:** conceptualization (lead), writing – original draft (lead), writing – review and editing (lead). **Sureshbabu Anjana:** writing – review and editing (supporting). **Do Thi Cat Tuong:** writing – review and editing (supporting). **Sungyeon Chin:** writing – review and editing (supporting). **Mohammad Moniruzzaman:** writing – review and editing (supporting). **Bui Huy Doanh:** writing – review and editing (supporting). **Adhimoolam Karthikeyan:** conceptualization (equal), project administration (equal), supervision (equal), writing – review and editing (supporting). **Taesun Min:** conceptualization (equal), funding acquisition (equal), project administration (equal), writing – review and editing (supporting).

## Ethics Statement

The authors have nothing to report.

## Consent

The authors have nothing to report.

## Conflicts of Interest

The authors declare no conflicts of interest.

## Data Availability

The authors have nothing to report.
